# Briarane Diterpenoids Isolated from Gorgonian Corals between 2011 and 2013

**DOI:** 10.3390/md12042164

**Published:** 2014-04-10

**Authors:** Jyh-Horng Sheu, Yung-Husan Chen, Yu-Hsin Chen, Yin-Di Su, Yu-Chia Chang, Jui-Hsin Su, Ching-Feng Weng, Chia-Hung Lee, Lee-Shing Fang, Wei-Hsien Wang, Zhi-Hong Wen, Yang-Chang Wu, Ping-Jyun Sung

**Affiliations:** 1 Department of Marine Biotechnology and Resources and Asia-Pacific Ocean Research Center, National Sun Yat-sen University, Kaohsiung 804, Taiwan; E-Mails: sheu@mail.nsysu.edu.tw (J.-H.S.); gobetter04@yahoo.com.tw (Y.-D.S.); whw@nmmba.gov.tw (W.-H.W.); wzh@mail.nsysu.edu.tw (Z.-H.W.); 2 Doctoral Degree Program in Marine Biotechnology, National Sun Yat-sen University and Academia Sinica, Kaohsiung 804, Taiwan; E-Mail: jay0404@gmail.com; 3 National Museum of Marine Biology and Aquarium, Pingtung 944, Taiwan; E-Mails: tony_chen72001@yahoo.com.tw (Y.-H.C.); kb5634@yahoo.com.tw (Y.-H.C.); x2219@nmmba.gov.tw (J.-H.S.); 4 Graduate Institute of Marine Biotechnology, Department of Life Science and Institute of Biotechnology, National Dong Hwa University, Pingtung 944, Taiwan; E-Mails: cfweng@mail.ndhu.edu.tw (C.-F.W.); chlee016@mail.ndhu.edu.tw (C.-H.L.); 5 Department of Sport, Health, and Leisure, Cheng Shiu University, Kaohsiung 833, Taiwan; E-Mail: lsfang@csu.edu.tw; 6 School of Pharmacy, College of Pharmacy, China Medical University, Taichung 404, Taiwan; 7 Chinese Medicine Research and Development Center, China Medical University Hospital, Taichung 404, Taiwan; 8 Center for Molecular Medicine, China Medical University Hospital, Taichung 404, Taiwan; 9 Graduate Institute of Natural Products, Kaohsiung Medical University, Kaohsiung 807, Taiwan

**Keywords:** Gorgonacea, briarane, *Briareum*, *Dichotella*, *Junceella*, *Verrucella*

## Abstract

The structures, names, bioactivities and references of 138 briarane-type diterpenoids, including 87 new compounds, are summarized in this review. All the briarane-type compounds mentioned in this review article were obtained from gorgonian corals including the genus *Briareum*, *Dichotella*, *Junceella* and *Verrucella*. Some of these compounds showed potential bioactivities.

## 1. Introduction

**Scheme 1:**
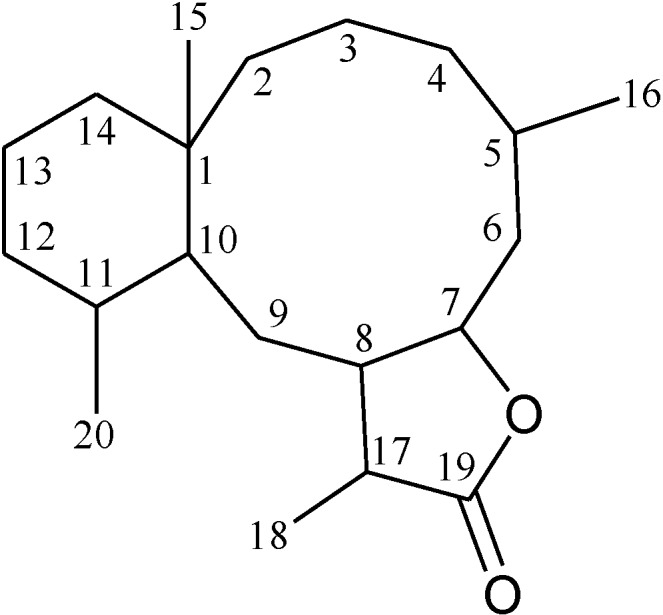
The carbon skeleton of briarane-type compounds.

This review describes the structures, names, bioactivities and references for all diterpenoid compounds in tabular form. This study reviewed literature from 2011 to 2013 and describes 138 briarane-type diterpenoids (including 87 new compounds) that possess a bicycle [8.4.0] carbon skeleton, and most possess a γ-lactone moiety in their structure (Scheme 1). As in previous reviews [1,2,3,4], we showed the structures, names, bioactivities and references for these briaranes. All briaranes mentioned in this article were isolated from octocorals belonging to the order Gorgonacea, including *Briareum asbestinum*, *Briareum excavatum*, *Briareum* spp., *Dichotella fragilis*, *Dichotella gemmacea*, *Junceella fragilis*, *Junceella juncea* and *Verrucella umbraculum*. This survey of briarane-type compounds is presented taxonomically according to genus and species.

## 2. Gorgonacea

### 2.1. Genus Briareum (Family Briareidae)

#### 2.1.1. *Briareum asbestinum*

Gorgonian corals belonging to the genus *Briareum* play a main role in producing briarane-type natural products. In further studies on the chemical constituents of Caribbean gorgonian *B. asbestinum*, the most famous species related to briarane metabolites, collected at Hillsboro Ledge, Boca Raton, Florida, yielded nine briareolate ester metabolites, including five new compounds, briareolate esters J–N (**1**–**5**) (Table 1) [5,6], and four known analogues, briareolate esters B–D and G [5,6,7,8]. Briareolate esters are a unique group of briaranes that contain a C-19 methyl ester instead of the γ-lactone ring, and compounds of this type have only been found in *B. asbestinum*. Briaranes **3** and **4** have been proven to be the first natural products possessing a 10-membered ring with an (*E*,*Z*)-dieneone moiety, and they exhibit cytotoxicity towards BG02 and BxPC-3 cells. SAR (structure-activity-relationship) study confirmed the importance of the (*E*,*Z*)-dieneone moiety for bioactivity among briaranes **1**–**4** [5].

**Table 1:** New briaranes from *B. asbestinum*.

Structure	No.	Name	Bioactivity	Ref.
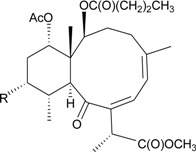	**1**	Briareolate ester J (R = OC(O)(CH_2_)_4_CH_3_)		[5]
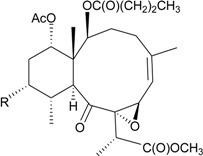	**2**	Briareolate ester K (R = OC(O)(CH_2_)_4_CH_3_)	EC_50_ (BG02) = 40 μM	[5]
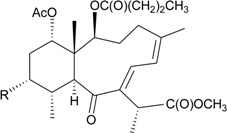	**3**	Briareolate ester L (R = OH)	EC_50_ (BG02, BxPC-3) *^a^* = 2.4, 9.3 μM	[6]
	**4**	Briareolate ester M (R = OC(O)(CH_2_)_4_CH_3_)	EC_50_ (BG02) = 8.0 μM briarane **4** showed cytostatic effects at 13.0 and 17.0 μM against the BxPC-3 cells	[6]
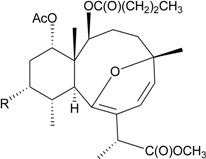	**5**	Briareolate ester N (R = OC(O)(CH_2_)_4_CH_3_)		[6]

*^a^* BG02 (human embryonic stem cell line), BxPC-3 (human pancreatic cancer cell line).

#### 2.1.2. *Briareum excavatum*

In continuation of the search for new natural products from marine invertebrates collected off the waters of Taiwan at the intersection of the Kuroshio current and the South China Sea surface current, gorgonian *B. excavatum*, collected at Orchid Island off Taiwan, was examined for its complex and interesting chemical constituents. Eight briarane derivatives, including six new compounds, briacavatolides A–F (**6**–**11**) [9,10] (Table 2) and two known metabolites, briaexcavatolide U and briaexcavatin L [9,11,12], were isolated. Briacavatolides C (**8**) and F (**11**) were found to show antiviral activity against HCMV using a human embryonic lung (HEL) cell line [9,10]. By comparing the structures of **8** and **10**, the 9-acetoxy group was found to be essential for the anti-HCMV activity by SAR study.

**Table 2:** New briaranes from *B. excavatum*.

Structure	No.	Name	Bioactivity	Ref.
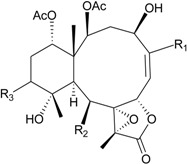	**6**	Briacavatolide A (R_1_ = CH_3_, R_2_ = OH, R_3_ = β-OH)	-	[9]
	**7**	Briacavatolide B (R_1_ = CH_2_OAc, R_2_ = OAc, R_3_ = β-OH)	-	[9]
	**8**	Briacavatolide C (R_1_ = CH_3_, R_2_ = OAc, R_3_ = α-OC(O)(CH_2_)_2_CH_3_)	IC_50_ (HCMV) = 18 μM *^a^*	[9]
	**10**	Briacavatolide E (R_1_ = CH_3_, R_2_ = OH, R_3_ = α-OC(O)(CH_2_)_2_CH_3_)	-	[10]
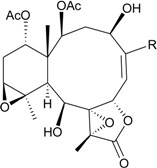	**9**	Briacavatolide D (R = CH_2_OH)	-	[10]
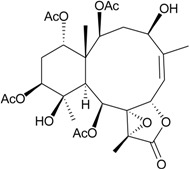	**11**	Briacavatolide F	IC_50_ (HCMV) = 22 μM	[10]

*^a^* HCMV (human cytomegalovirus).

#### 2.1.3. *Briareum* spp.

Brialalepolides A–C (**12**–**14**), three new briaranes, were isolated from gorgonian *Briareum* sp., collected in the Republic of Vanuatu [13] (Table 3). The structure, including the absolute configuration, of **12** was further confirmed by X-ray diffraction using the Hooft method [13]. Briaranes **12**–**14** exhibited dose-independent cytotoxicity against Caco-2 cells over a range of 5–30 μM. Briaranes **13** and **14** reduced the expression of COX-2 in Caco-2 and RAW 264.7 cells [13].

The organic extracts of gorgonian *Briareum* sp. collected from the coral reef of Ishigaki Island, Okinawa, Japan, were examined. Ten briarane metabolites, including seven new diepoxybriaranes, briaroxalides A–G (**15**–**21**) [14] (Table 3), along with three known analogues, brianthein A [15], violide G [16] and briarlide R [17], were isolated. The absolute configurations of **15**–**21** were further confirmed by chemical conversion and X-ray diffraction analysis [14].

Three new diterpenoids, briarenolides E–G (**22**–**24**) (Table 3), were isolated from gorgonian *Briareum* sp. collected off the coast of Southern Taiwan [18,19]. Compounds **22** and **23** were the first 2-ketobriarane and 6-hydroperoxybriarane diterpenoids, respectively. Briarane **23** displayed a significant inhibitory effect on the generation of superoxide anions by human neutrophils [19].

**Table 3:** New briaranes from *Briareum* spp.

Structure	No.	Name	Bioactivity	Ref.
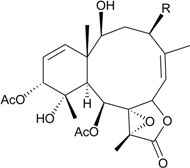	**12**	Brialalepolide A (R = OAc)	at concentrations of 30, 20 and 15 μM for compounds **12**–**14**, respectively, an approximately 50% decrease in cell viability on Caco-2 cells	[13]
	**13**	Brialalepolide B (R = OC(O)(CH_2_)_4_CH_3_)		[13]
	**14**	Brialalepolide C (R = OC(O)(CH_2_)_6_CH_3_)	compounds **13** and **14** reduced levels of COX-2 mRNA in Caco-2 and RAW 264.7 cells at concentrations of 10–15 μM *^a^*	[13]
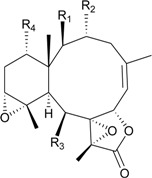	**15**	Briaroxalide A (R_1_ = R_3_ = OH, R_2_ = R_4_ = OAc)		[14]
	**16**	Briaroxalide B (R_1_ = R_2_ = OH, R_3_ = R_4_ = OAc)		[14]
	**17**	Briaroxalide C (R_1_ = OH, R_2_ = R_3_ = R_4_ = OAc)		[14]
	**18**	Briaroxalide D (R_1_ = R_2_ = R_4_ = OH, R_3_ = OAc)		[14]
	**19**	Briaroxalide E (R_1_ = R_4_ = OH, R_2_ = R_3_ = OAc)		[14]
	**20**	Briaroxalide F (R_1_ = R_2_ = R_3_ = OAc, R_4_ = OH)		[14]
	**21**	Briaroxalide G (R_1_ = R_3_ = R_4_ = OAc, R_2_ = OH)		[14]
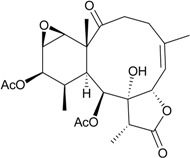	**22**	Briarenolide E	showed inhibitory effects on the generation of superoxide anion (inhibition rate = 23.7%) and release of elastase (inhibition rate = 28.3%) at 10 μg/mL	[18]
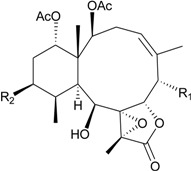	**23**	Briarenolide F (R_1_ = OOH, R_2_ = OC(O)(CH_2_)_2_CH_3_)	showed inhibitory effects on the generation of superoxide anion (inhibition rate = 76.7%) (IC_50_ = 3.8 μg/mL) and release of elastase (inhibition rate = 27.5%) at 10 μg/mL	[19]
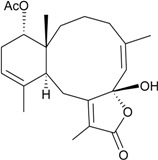	**24**	Briarenolide G	showed inhibitory effects on the generation of superoxide anion (inhibition rate = 22.0%) and release of elastase (inhibition rate = 13.0%) at 10 μg/mL	[19]

*^a^* COX-2 (cyclooxygenase-2 or prostaglandin-endoperoxide synthase 2), Caco-2 (human epithelial colon adenocarcinoma), RAW264.7 (mouse leukemic monocyte macrophage cell line).

### 2.2. Genus Dichotella (Family Ellisellidae)

#### 2.2.1. *Dichotella fragilis*

Four known briaranes, juncins P and U [20,21], junceellolide D [22] and junceol A [23], were isolated from gorgonian *D. fragilis*, collected from Meishan, Sanya sea area in the South China Sea [24]. Juncin P and junceellolide D were found to display antifouling activity against the larval settlement of the barnacle *Balanus amphitrite*, with EC_50_ values of 0.80 and 0.77 μg/mL, respectively [24]. Juncin U displayed mild antifouling activity against the larval settlement of *B. amphitrite* at a concentration of 50.0 μg/mL [24].

#### 2.2.2. *Dichotella gemmacea*

In the past three years, the gorgonian coral *D. gemmacea*, collected from the South China Sea, has been proven to be a rich source of novel briarane-type metabolites. Thirty-six new briaranes, gemmacolides G (**25**), I–Y (**26**–**42**) and AA–AR (**43**–**60**) [25,26,27,28] (Table 4), along with known briaranes, juncins O, R, S, U [20,21], junceellolide C [22], 12-*epi*-fragilide G [29,30], juncenolides C, D, J [31,32] and praelolide [33,34], were isolated from *D. gemmacea* by Zhang’s group [25,26,27,28]. By NOESY experiments, the diene system between C-3/4 and C-5/16 in **25**–**27** was established as a conjugated *s-cis* diene moiety [29,30]. The absolute configurations of **31**–**60** were elucidated by TDDFT calculation of their solution ECD spectrum and by biosynthetic consideration [26,27,28].

New briaranes **25**–**28**, **30**, **34**–**40**, **42**–**45**, **47**, **48**, **50**–**54** and **56**–**60** exhibited different levels of cytotoxicity against A549 and MG63 cells [25,26,27,28]. Cytotoxicities of known briaranes 12-*epi*-fragilide G, juncins R, S and U, juncenolides D and J and praelolide towards A549 (IC_50_ = 47.3, 13.9, 20.2, >43.2, 37.1, >46.7, >50.1 μM) and MG63 cells (IC_50_ = 54.0, 5.6, 16.5, >43.2, >46.0, >46.7, >50.1 μM) were also reported [25,26,27].

It is interesting to note that gemmacolides J (**27**), V (**39**) and Y (**42**) showed significant activities towards A549 cells as compared with the activities of their analogues and the positive control (doxorubicin, IC_50_ = 2.8 μM) [25,27]. Gemmacolide Y (**42**) displayed stronger activity toward MG63 cells than its analogues and the positive control (doxorubicin, IC_50_ = 3.2 μM) [27]. SAR study of the active components **27**, **39** and **42** and their analogues will potentially lead to the discovery of agents of medical benefit.

In antimicrobial tests, briarane **27** exhibited antimicrobial activity against the bacterium *Bacillus megaterium* [25]. Briaranes **31**, **32**, **34** and **37**–**42** exhibited activity against the bacterium *Escherichia coli* [26,27]. Briaranes **30**, **31**, **34** and **37**–**42** displayed antifungal activity against *Septoria tritici* [25,26,27]. Briaranes **37**–**42** exhibited activity against the fungus *Microbotryum violaceum* [27]. Known briaranes juncenolides D and J, juncins R, S and U and praelolide exhibited antibacterial and antifungal activity against the bacterium *E. coli* (*Ф* = 12.5, 11.0, 14.0, 10.0, 11.0, 18.0 mm) and the fungus *S. tritici* (*Ф* = 7.5, 12.0, 7.5, 7.0, 7.5, 15.0 mm) [26,27]. Juncenolide J and praelolide were also found to display antifungal activity against *M. violaceum* (*Ф* = 10.0, 11.0 mm) [27].

Furthermore, 15 new briarane derivatives, dichotellides F–S (**61**–**74**) and U (**75**) [35], along with a series of known metabolites, juncenolide D [31], gemmacolide N [26], juncins D, P, Q, Y and ZI [20,21,36], praelolide [33,34], junceellolides C and D [22], (+)-11β,12β-epoxyjunceellolide D [37], dichotellides A–E [38], junceellin A [34,39,40] and gemmacolide X [27,35], were obtained from *D. gemmacea*, collected from Meishan Island, Hainan province of China, by Liu’s group [35]. The structure, including the absolute configuration, of gemmacolide X was further confirmed by single-crystal X-ray diffraction data analysis [35]. The structure of praelolide shown in this article was duplicated. The structure of dichotellide T was found to be identical as that of gemmacolide X, a briarane previously reported in ref. [27].

In the antifouling activity test, briaranes **63**, **64**, **66**–**71** and **75** showed potent antifouling activities at nontoxic concentrations against the larval settlement of barnacle *B. amphitrite*. Known briarane junceellolide C showed a significant inhibitory effect on larval settlement at a concentration of 5.0 μg/mL (EC_50_ = 0.2 μg/mL, LC_50_/EC_50_ > 500) [35]. Briaranes **64**, **69**, **71**, **75** and junceellolide C have high therapeutic ratios (LC_50_/EC_50_), suggesting that these compounds might be useful as environmentally benign antifouling agents [35]. SAR study of the active components **63**, **64** and **66**–**71** and **75** and their analogues has been performed [35].

**Table 4:** New briaranes from *D. gemmacea*.

Structure	No.	Name	Bioactivity	Ref.
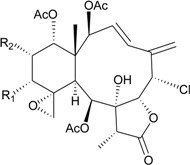	**25**	Gemmacolide G (R_1_ = R_2_ = OAc)	IC_50_ (A549, MG63) *^a^* = 8.4, 38.4 μM	[25]
	**26**	Gemmacolide I (R_1_ = OC(O)CH_2_CH(CH_3_)_2_, R_2_ = H)	IC_50_ (A549, MG63) = 20.6, 25.0 μM	[25]
	**27**	Gemmacolide J (R_1_ = OC(O)CH_2_CH(CH_3_)_2_, R_2_ = OAc)	IC_50_ (A549, MG63) ≤1.4, 79.8 μM briarane **27** exhibited antibacterial activity against *B. megaterium* (*Ф* = 16.0 mm)	[25]
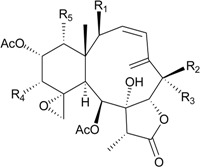	**28**	Gemmacolide K (R_1_ = OC(O)CH_2_OH, R_2_ = H, R_3_ = Cl, R_4_ = OC(O)CH_2_CH(CH_3_)_2_, R_5_ = OAc)	IC_50_ (A549, MG63) = 38.2, 45.9 μM	[25]
	**29**	Gemmacolide L (R_1_ = OC(O)CH_2_OC(O)CH_2_CH(CH_3_)_2_, R_2_ = H, R_3_ = Cl, R_4_ = OC(O)CH_2_CH(CH_3_)_2_, R_5_ = OAc)	-	[25]
	**30**	Gemmacolide M (R_1_ = R_4_ = OAc, R_2_ = OCH_3_, R_3_ = H, R_5_ = OC(O)CH_2_CH(CH_3_)_2_)	IC_50_ (A549) = 27.4 μM exhibited antifungal activity against *S. tritici* (*Ф* = 15.0 mm)	[25]
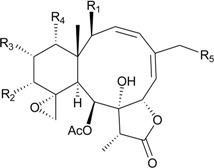	**31**	Gemmacolide N (R_1_ = R_2_ = R_4_ = OAc, R_3_ = H, R_5_ = OCH_3_)	briaranes **31**–**36** exhibited cytotoxicity toward A549 (IC_50_ ≥ 50.5, >44.6, >44.1, 21.6, 27.2, 16.4 μM) and MG63 (IC_50_ ≥ 50.5, >44.6, >44.1, 20.5, 23.7, 18.8 μM) cells briaranes 31, 32 and 34 exhibited antibacterial activity against *E. coli* (*Ф* = 12.5, 13.0, 10.0 mm)	[26]
	**32**	Gemmacolide O (R_1_ = OC(O)CH_2_OH, R_2_ = R_3_ = R_4_ = OAc, R_5_ = Cl)		[26]
	**33**	Gemmacolide P (R_1_ = R_3_ = R_4_ = OAc, R_2_ = OC(O)CH_2_CH(CH_3_)_2_, R_5_ = OH)		[26]
	**34**	Gemmacolide Q (R_1_ = OC(O)CH_2_OH, R_2_ = OC(O)CH_2_CH(CH_3_)_2_, R_3_ = R_4_ = OAc, R_5_ = OH)		[26]
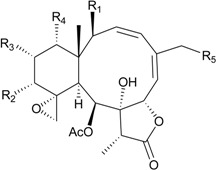	**35**	Gemmacolide R (R_1_ = OC(O)CH_2_OH, R_2_ = R_3_ = OAc, R_4_ = OC(O)CH_2_CH(CH_3_)_2_, R_5_ = OH	briaranes **31**, **32** and **34** exhibited antibacterial activity against *E. coli* (*Ф* = 12.5, 13.0, 10.0, mm) briaranes 31 and 34 exhibited antifungal activity against *S. tritici* (*Ф* = 7.5, 7.5 mm) briaranes **43**–**45**, **47**, **48**, **50**–**52**, **54** and **56**–**60** exhibited cytotoxicity toward A549 cells (IC_50_ = 14.7, 19.4, 17.9, 20.1, 27.4, 5.0, 27.7, 39.9, >37.8, 13.4, 78.5, 10.1, 28.7, 16.8 μM) briaranes **43**–**45**, **47**, **48**, **50**–**54** and **56**–**59** exhibited cytotoxicity toward MG63 cells (IC_50_ = 28.7, 22.8, 42.7, 41.3, 33.0, 5.0, 37.5, 9.1, 39.0, 37.8, 12.1, 25.8, 17.1, >100 μM)	[26]
	**36**	Gemmacolide S (R_1_ = OC(O)CH_2_OC(O)CH_2_CH(CH_3_)_2_, R_2_ = R_4_ = OAc, R_3_ = R_5_ = OC(O)CH_2_CH(CH_3_)_2_)		[26]
	**43**	Gemmacolide AA (R_1_ = OC(O)CH_2_OH, R_2_ = R_3_ = R_4_ = OAc, R_5_ = OCH_3_)		[28]
	**44**	Gemmacolide AB (R_1_ = OC(O)CH_2_OH, R_2_ = OC(O)CH_2_CH(CH_3_)_2_, R_3_ = R_4_ = OAc, R_5_ = OCH_3_)		[28]
	**45**	Gemmacolide AC (R_1_ = R_3_ = R_4_ = OAc, R_2_ = OC(O)CH_2_CH(CH_3_)_2_, R_5_ = OCH_3_)		[28]
	**46**	Gemmacolide AD (R_1_ = R_3_ = OAc, R_2_ = R_4_ = OC(O)CH_2_CH(CH_3_)_2_, R_5_ = OCH_3_)		[28]
	**47**	Gemmacolide AE (R_1_ = OC(O)CH_2_OC(O)CH_2_CH(CH_3_)_2_, R_2_ = R_3_ = H, R_4_ = OAc, R_5_ = OCH_3_)		[28]
	**48**	Gemmacolide AF (R_1_ = R_3_ = R_4_ = OAc, R_2_ = R_5_ = OC(O)CH_2_CH(CH_3_)_2_)		[28]
	**49**	Gemmacolide AG (R_1_ = R_2_ = R_3_ = R_4_ = OAc, R_5_ = OC(O)CH_2_CH(CH_3_)_2_)		[28]
	**50**	Gemmacolide AH (R_1_ = OC(O)CH_2_OC(O)CH_2_CH(CH_3_)_2_, R_2_ = R_5_ = OC(O)CH_2_CH(CH_3_)_2_, R_3_ = R_4_ = OAc)		[28]
	**51**	Gemmacolide AI (R_1_ = OC(O)CH_2_OC(O)CH_2_CH(CH_3_)_2_, R_2_ = OH, R_3_ = R_4_ = OAc, R_5_ = OC(O)CH_2_CH(CH_3_)_2_)		[28]
	**52**	Gemmacolide AJ (R_1_ = OC(O)CH_2_OC(O)CH_2_CH(CH_3_)_2_, R_2_ = OC(O)CH_2_CH(CH_3_)_2_, R_3_ = R_4_ = OAc, R_5_ = Cl)		[28]
	**53**	Gemmacolide AK (R_1_ = OC(O)CH_2_OH, R_2_ = R_4_ = OAc, R_3_ = OC(O)CH_2_CH(CH_3_)_2_, R_5_ = OCH_3_)		[28]
	**54**	Gemmacolide AL (R_1_ = OC(O)CH_2_OC(O)CH_2_CH(CH_3_)_2_, R_2_ = R_4_ = OAc, R_3_ = OC(O)CH_2_CH(CH_3_)_2_, R_5_ = OCH_3_)		[28]
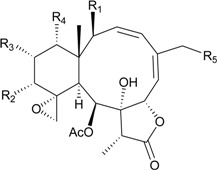	**55**	Gemmacolide AM (R_1_ = OC(O)CH_2_OC(O)CH_2_CH(CH_3_)_2_, R_2_ = R_4_ = OAc, R_3_ = OC(O)CH_2_CH(CH_3_)_2_, R_5_ = Cl)	briaranes **63**, **64**, **66**–**71** and **75** showed antifouling activity against the larval settlement of barnacle *B. amphitrite* (EC_50_ = 4.1, 1.82, 6.3, 7.6, 4.6, 1.2, 5.6, 0.70, 2.0 μg/mL)	[28]
	**56**	Gemmacolide AN (R_1_ = OC(O)CH_2_OH, R_2_ = R_4_ = OAc, R_3_ = OC(O)CH_2_CH(CH_3_)_2_, R_5_ = Cl)		[28]
	**57**	Gemmacolide AO (R_1_ = OC(O)CH_2_OH, R_2_ = R_3_ = R_5_ = OC(O)CH_2_CH(CH_3_)_2_, R_4_ = OAc)		[28]
	**58**	Gemmacolide AP (R_1_ = OC(O)CH_2_OH, R_2_ = R_3_ = OAc, R_4_ = OC(O)CH_2_CH(CH_3_)_2_, R_5_ = Cl)		[28]
	**59**	Gemmacolide AQ (R_1_ = R_2_ = R_3_ = OAc, R_4_ = OC(O)CH_2_CH(CH_3_)_2_, R_5_ = OH)		[28]
	**60**	Gemmacolide AR (R_1_ = R_2_ = R_3_ = R_5_ = OAc, R_4_ = OC(O)CH_2_CH(CH_3_)_2_)		[28]
	**61**	Dichotellide F (R_1_ = OC(O)CH_2_OC(O)CH_2_CH(CH_3_)_2_, R_2_ = R_3_ = R_4_ = OAc, R_5_ = OCH_3_)		[35]
	**62**	Dichotellide G (R_1_ = OC(O)CH_2_OC(O)CH_2_CH(CH_3_)_2_, R_2_ = R_4_ = OAc, R_3_ = OC(O)CH_2_CH(CH_3_)_2_, R_5_ = OCH_3_)		[35]
	**63**	Dichotellide H (R_1_ = OC(O)CH_2_OC(O)CH_2_CH(CH_3_)_2_, R_2_ = OC(O)CH_2_CH(CH_3_)_2_, R_3_ = R_4_ = OAc, R_5_ = Cl)		[35]
	**64**	Dichotellide I (R_1_ = OC(O)CH_2_OC(O)CH_2_CH(CH_3_)_2_, R_2_ = R_3_ = R_4_ = OAc, R_5_ = OC(O)CH_2_CH(CH_3_)_2_)		[35]
	**65**	Dichotellide J (R_1_ = R_2_ = R_3_ = R_4_ = R_5_ = OAc)		[35]
	**66**	Dichotellide K (R_1_ = R_2_ = R_4_ = OAc, R_3_ = R_5_ = OC(O)CH_2_CH(CH_3_)_2_)		[35]
	**67**	Dichotellide L (R_1_ = R_2_ = R_3_ = OAc, R_4_ = R_5_ = OC(O)CH_2_CH(CH_3_)_2_)		[35]
	**68**	Dichotellide M (R_1_ = R_4_ = OAc, R_2_ = R_3_ = OC(O)CH_2_CH(CH_3_)_2_, R_5_ = OCH_3_)		[35]
	**69**	Dichotellide N (R_1_ = R_2_ = OAc, R_3_ = H, R_4_ = OC(O)CH_2_CH(CH_3_)_2_, R_5_ = OCH_3_)		[35]
-	**70**	Dichotellide O (R_1_ = R_4_ = OAc, R_2_ = OC(O)CH_2_CH(CH_3_)_2_, R_3_ = H, R_5_ = OCH_3_)	-	[35]
	**71**	Dichotellide P (R_1_ = R_2_ = OAc, R_3_ = H, R_4_ = R_5_ = OC(O)CH_2_CH(CH_3_)_2_)		[35]
	**72**	Dichotellide Q (R_1_ = OAc, R_2_ = R_4_ = OH, R_3_ = OC(O)CH_2_CH(CH_3_)_2_, R_5_ = Cl)		[35]
	**73**	Dichotellide R (R_1_ = OAc, R_2_ = R_4_ = OH, R_3_ = R_5_ = OC(O)CH_2_CH(CH_3_)_2_)		[35]
	**74**	Dichotellide S (R_1_ = R_3_ = OAc, R_2_ = R_4_ = OH, R_5_ = OC(O)CH_2_CH(CH_3_)_2_)		[35]
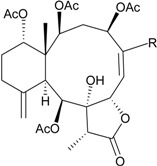	**75**	Dichotellide U (R = CH_2_OC(O)CH_2_CH(CH_3_)_2_)		[35]
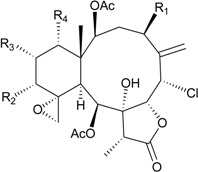 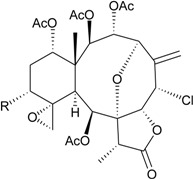	**37**	Gemmacolide T (R_1_ = R_4_ = OAc, R_2_ = OC(O)CH_2_CH(CH_3_)_2_, R_3_ = H)	briaranes **37**–**42** exhibited cytotoxicity toward A549 (IC_50_ = 16.9, 18.0, <1.5, 19.1, >45.7, <0.3 μM) and MG63 (IC_50_ = 18.0, 15.1, 20.5, 17.4, >45.7, <0.3 μM) cells briaranes **37**–**42** exhibited antibacterial activity against *E. coli* (*Ф* = 19.0, 20.0, 20.0, 17.0, 20.0, 34.0 mm) briaranes **37**–**42** exhibited antifungal activity against *M. violaceum* (*Ф* = 14.0, 9.5, 11.0, 13.0, 15.0, 11.0 mm) and *S. tritici* (*Ф* = 14.0, 9.5, 13.0, 17.0, 12.0, 13.0 mm)	[27]
	**38**	Gemmacolide U (R_1_ = R_2_ = OAc, R_3_ = H, R_4_ = OC(O)CH_2_CH(CH_3_)_2_)		[27]
	**39**	Gemmacolide V (R_1_ = R_2_ = R_4_ = OAc, R_3_ = H)		[27]
	**40**	Gemmacolide W (R_1_ = R_3_ = OC(O)CH_2_CH(CH_3_)_2_, R_2_ = R_4_ = OAc)		[27]
	**41**	Gemmacolide X (R = OAc) (=Dichotellide T)		[27,35]
	**42**	Gemmacolide Y (R = OC(O)CH_2_CH(CH_3_)_2_)		[27]

*^a^* A549 (human lung epithelial carcinoma), MG63 (human osteosarcoma).

### 2.3. Genus Junceella (Family Ellisellidae)

#### 2.3.1. *Junceella fragilis*

Studies of the gorgonian coral *J. fragilis*, collected off the south-eastern Taiwan coast, have afforded eight new briaranes, frajunolides L–S (**76**–**83**) [41,42] (Table 5). The structure of frajunolide P (**80**) was further confirmed by X-ray crystallographic data analysis [42]. Briaranes **76**–**81** exhibited inhibitory effects on the generation of superoxide anions and the release of elastase by human neutrophils [41,42].

**Table 5:** New briaranes from *J. fragilis*.

Structure	No.	Name	Bioactivity	Ref.
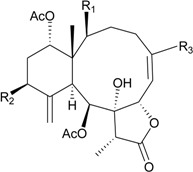	**76**	Frajunolide L (R_1_ = R_2_ = OAc, R_3_ = CH_3_)	briaranes **76**, **80** and **81** showed inhibitory effects on the generation of superoxide anion (inhibition rate = 18.7%, 32.5%, 28.7%) and the release of elastase (inhibition rate = 16.2%, 35.6%, 34.1%) at 10 μg/mL	[41]
	**80**	Frajunolide P (R_1_ = OC(O)C(CH_3_)_3_, R_2_ = H, R_3_ = C(O)OCH_3_)		[42]
	**81**	Frajunolide Q (R_1_ = OAc, R_2_ = H, R_3_ = C(O)OCH_3_)		[42]
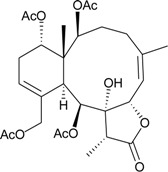	**77**	Frajunolide M	briaranes **77**–**79** and **82** showed inhibitory effects on the release of elastase (inhibition rate = 13.1%, 22.3%, 17.2%, 16.0%) at 10 μg/mL	[41]
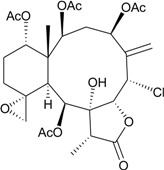	**78**	Frajunolide N	-	[41]
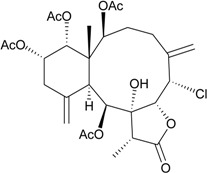	**79**	Frajunolide O	-	[41]
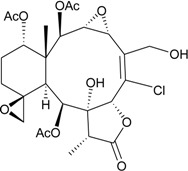	**82**	Frajunolide R	-	[42]
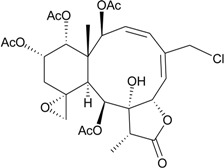	**83**	Frajunolide S	-	[42]

#### 2.3.2. *Junceella juncea*

Murthy *et al.* [43] isolated a new 8-hydroxybriarane, **84** (Table 6), along with four known analogues, gemmacolides A and B [44] and juncins H and K [45,46], from the gorgonian coral *Junceella juncea* [43], collected from Tuticorin coast of the Indian Ocean. Briarane **84** showed moderate activities against the fungi *Aspergillus niger*, *Candida albicans* and *Penicillium notatum*. The known isolates exhibited activities against various bacteria [43]. Furthermore, Shen *et al.* [47] isolated three new briaranes, juncenolides M–O (**85**–**87**), from *J. juncea*, collected in the waters of Taiwan. Briaranes **85**–**87** exhibited inhibitory effects on the generation of superoxide anions and the release of elastase by human neutrophils [47].

**Table 6:** New briarane metabolites from *J. juncea*.

Structure	No.	Name	Bioactivity	Ref.
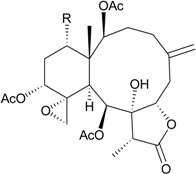	**84**	R = OC(O)CH_2_CH(CH_3_)_2_ *^a^*	showed moderate activity against the fungi *Aspergillus niger*, *Candida albicans* and *Penicillium notatum* (inhibition zone = 18, 17, 16 mm)	[43]
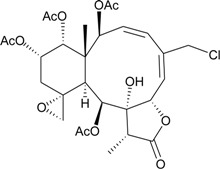	**85**	Juncenolide M	briaranes **85**–**87** showed inhibitory effects on the release of elastase (inhibition rate = 15.9%, 29.0%, 35.9%) at 10 μg/mL	[47]
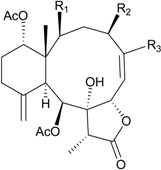	**86**	Juncenolide N (R_1_ = OH, R_2_ = H, R_3_ = CH_3_)	-	[47]
	**87**	Juncenolide O (R_1_ = R_2_ = OAc, R_3_ = C(O)OCH_3_)	showed an inhibitory effect on the generation of superoxide anion (inhibition rate = 27.6%) at 10 μg/mL	[47]

*^a^* This compound was named (1S*,2S*,8S*,9S*,10S*,11R*,12R*,14S*,17R*)-11,20-epoxy-14-(3-methylbutanoyl)-2,9,12,-triacetoxy-8-hydroxybriar-5(16)-en-18,7-olide. Because the stereochemistry of C-7 chiral carbon was not described, the stereochemistry of C-7 was assigned as S*-configuration by the structure of **84** [43].

### 2.4. Genus Verrucella (Family Ellisellidae)

#### 2.4.1. *Verrucella umbraculum*

Six known briaranes, robustolide A [48], renillafoulin A [49], erythrolide B [50], (–)-4-deacetyljunceellolide D [37], junceellonoid D [51] and frajunolide A [52], were claimed to have been obtained from gorgonian coral *Verrucella umbraculum* [53]. However, through detailed analysis, the NMR data of all compounds reported in this study were substantially different to data reported previously [37,48,49,50,51,52]. The authors suggested that the compounds described in this paper should be re-examined.

## 3. Conclusions

In 1977, the first briarane-type natural product, briarein A, was isolated from the Caribbean gorgonian *Briareum asbestinum* [54]. To date, approximately 600 briarane-type diterpenoids have been isolated from various marine organisms, particularly soft corals belonging to the order Gorgonacea. Compounds of this type of diterpenoid have been proven to possess various bioactivities. Except for the briaranes from *B. asbestinum*, all the briaranes reported between 2011 and 2013 were obtained from the gorgonian corals distributed in the Indo-Pacific Ocean, particularly from the South China Sea. Because of the structural complexity of the compounds, it is difficult to establish a stable supply of bioactive briaranes by chemical methods. Due to the potential medicinal applications, coral aquaculture to support bioactive briaranes is becoming very attractive [55,56,57,58,59]. For example, briaranes from *Briareum excavatum*, collected off the waters of Taiwan, were proven to possess significant anti-inflammatory activity [60], and in order to establish a stable supply of bioactive materials, the coral has been cultured successfully using a flow-through sea water system, in the National Museum of Marine Biology and Aquarium, Taiwan for the extraction of additional natural products.
